# A Hadal *Streptomyces*-Derived Echinocandin Acylase Discovered through the Prioritization of Protein Families

**DOI:** 10.3390/md22050212

**Published:** 2024-05-07

**Authors:** Xuejian Jiang, Hongjun Shu, Shuting Feng, Pinmei Wang, Zhizhen Zhang, Nan Wang

**Affiliations:** 1 Ocean College, Zhejiang University, Zhoushan 316021, China; xuejian_jiang@zju.edu.cn (X.J.); shuhj@zju.edu.cn (H.S.); 22234180@zju.edu.cn (S.F.); wangpinmei@zju.edu.cn (P.W.); zzhang88@zju.edu.cn (Z.Z.); 2 Hainan Institute of Zhejiang University, Sanya 572025, China

**Keywords:** echinocandin acylase, hadal bacterium, antifungal drugs, enzyme similarity tool, the Mariana Trench

## Abstract

Naturally occurring echinocandin B and FR901379 are potent antifungal lipopeptides featuring a cyclic hexapeptide nucleus and a fatty acid side chain. They are the parent compounds of echinocandin drugs for the treatment of severe fungal infections caused by the *Candida* and *Aspergilla* species. To minimize hemolytic toxicity, the native fatty acid side chains in these drug molecules are replaced with designer acyl side chains. The deacylation of the *N*-acyl side chain is, therefore, a crucial step for the development and manufacturing of echinocandin-type antibiotics. Echinocandin E (ECE) is a novel echinocandin congener with enhanced stability generated via the engineering of the biosynthetic machinery of echinocandin B (ECB). In the present study, we report the discovery of the first echinocandin E acylase (ECEA) using the enzyme similarity tool (EST) for enzymatic function mining across protein families. ECEA is derived from *Streptomyces* sp. SY1965 isolated from a sediment collected from the Mariana Trench. It was cloned and heterologously expressed in *S. lividans* TK24. The resultant TKecea66 strain showed efficient cleavage activity of the acyl side chain of ECE, showing promising applications in the development of novel echinocandin-type therapeutics. Our results also provide a showcase for harnessing the essentially untapped biodiversity from the hadal ecosystems for the discovery of functional molecules.

## 1. Introduction

Fungal infections can range from superficial conditions to life-threatening systemic infections. Echinocandins, including caspofungin, micafungin, anidulafungin and rezafungin, are new antifungal drugs, targeting the biosynthesis of β-(1,3)-d-glucan, an essential component of the fungal cell wall [1]. Since β-(1,3)-d-glucan is absent in mammalian cells, echinocandins act selectively against fungal cells [2]. The high efficacy and selectivity of echinocandins permits their frequent recommendations as the first-line drugs for many fungal infections, especially for invasive candidiasis and invasive aspergillosis [3]. 

**Figure 1:**
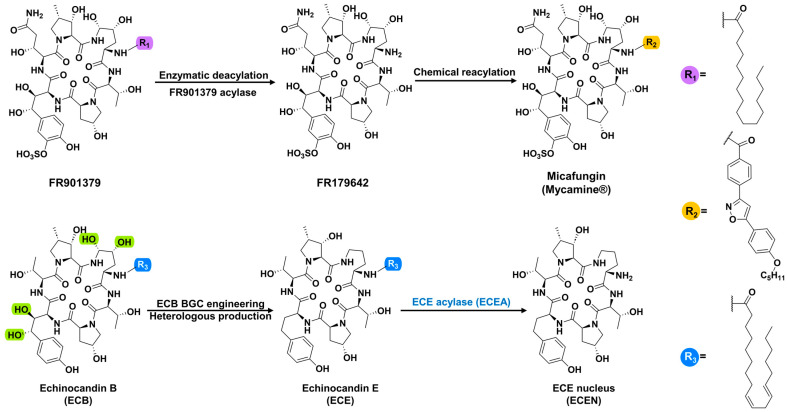
FR901379, as a representative echinocandin, shows the deacylation reaction catalyzed by FR901379 acylase and the chemical reacylation process to produce the antifungal drug micafungin (the upper arrow diagram); echinocandin E (ECE) is a novel tetradeoxy (green shade positions) analog of echinocandin B (ECB), generated using engineered ECB biosynthetic machinery. The deacylation of ECE by ECE acylase (ECEA) produces the ECE nucleus (ECEN) (the lower arrow diagram).

Echinocandins are semi-synthesized drugs derived from fungal natural products echinocandin B (ECB), pneumocandin B0, and FR901379. They are the parent compounds of anidulafungin (Eraxis^®^), caspofungin acetate (Cancidas^®^) micafungin (Mycamine^®^), and rezafungin (Rezzayo^®^) [4,5]. All of the three natural compounds have a cyclic hexapeptide core and a fatty acid side chain. While the native *N*-acyl fatty acid chain is crucial for inhibition of β-(1,3)-d-glucan synthase, it also causes hemolytic effects to the host cells. Consequently, a pivotal modification of naturally occurring echinocandins involves cleaving the *N*-linked acyl side chain to introduce customized acyl groups, thereby mitigating the lytic effect (Figure 1). Other minor chemical modifications on the hexapeptide nucleus are contributing to the improved solubility and stability of echinocandins.

*Actinoplanes utahensis*, or the acylases it produces, are usually used to hydrolyze the natural acyl side chain of echinocandins [6,7,8,9]. Several ECB acylases (also known as ECB deacylases, ECBDs) have been discovered for the enzymatic deacylation of leading compounds ECB and FR9013179 to produce corresponding nuclei (Figure 1) [6,7,9]. In our recent work, a novel echinocandin analog (echinocandin E, ECE) with an unprecedented tetradeoxy-hexapeptide nucleus was produced by heterologously expressing an engineered biosynthetic machinery of echinocandin B in *Aspergillus nidulans* [10]. While retaining the antifungal efficacy comparable with ECB, ECE displayed significantly enhanced chemical stability. Since considerable modifications have been introduced to the cyclic peptide core, there is no known catalyst available for the deacylation of this non-natural product ECE. Taking advantage of the structure similarity between ECB and ECE, we assume that ECBD [7] and the FR9013179 acylase [11] are also capable of hydrolyzing the *N*-attached side chain of ECE. Accordingly, they can be used as probes for the bioinformatic mining of new biocatalysts applicable to ECE deacylation.

Microbes found in the deep sea at hadal depths have adapted to extreme conditions. Hadal microorganisms often evolve unique biochemical pathways and metabolic capabilities that are of interest for biotechnological applications. The enzyme function initiative-enzyme similarity tool (EFI-EST) is a computational tool used for new enzymatic function discovery [12,13]. It employs algorithms and statistical methods to compare the sequences and quantify their degree of similarity [14]. The sequence similarity networks (SSNs) it generates, therefore, segregate proteins into discrete clusters according to their predicted functions [15]. In the present study, the EST-EFI tool was used for ECE-deacylating function mining. A hadal *Streptomyces*-derived echinocandin E acylase (ECEA) was successfully discovered, which demonstrated efficient deacylation activity toward ECE and FR9013179. Our findings unveil the inaugural identification of an ECE acylase, expecting to accelerate the drug development pipelines of ECE and other echinocandin-type antifungal reagents.

## 2. Results and Discussion

### 2.1. Hypothetic Echinocandin Acylases Prioritized by EFT-EST

**Figure 2:**
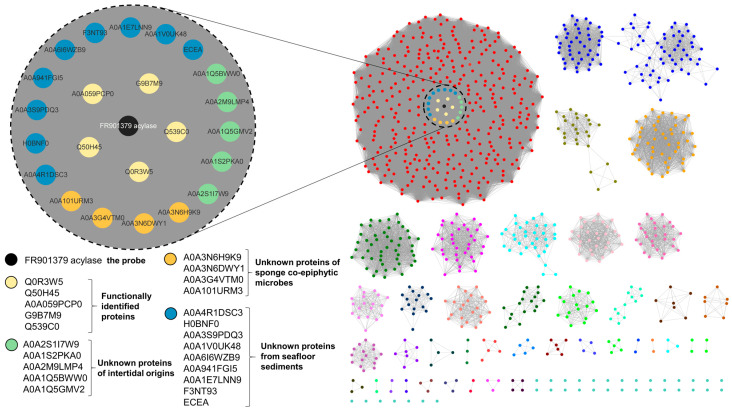
The sequence similarity network (SSN) generated using the EFI-EST web tool and visualized using Cytoscape, representing 987 protein homologs of the probe protein, FR901379 acylase. The zoomed insert on the upper left shows the proteins originating from marine sources (the brown, green and blue nodes) clustered with the probe (the black node) and the known proteins (the yellow nodes).

The SSN generated from 987 proteins comprises homologs of the probe (FR901379 acylase) [9] retrieved from the UniProt database and our in-house genomic sequence library of hadal bacteria. These proteins, used for the SNN calculation, are essentially from the Ntn-hydrolase superfamily of proteins, such as penicillin amidases/acylases (IPR043146/IPR043147/PF01804/IPR023343) and the *N*-terminal of the nucleophile aminohydrolases (IPR029055). Shown in Figure 2 is the SSN demonstrating sequence identities over 54.61% visualized with Cytoscape. FR901379 acylase clustered with three hundred and seventy-three protein entities, including five functionally characterized proteins, viz. an ECB acylase (G9B7M9), two penicillin V acylases (Q539C0 and Q0R3W5), and two acyl homoserine lactone acyltransferases (Q50H45 and A0A059PCP0). Of the unidentified proteins, 18 originated from marine sources (Table S1), including the hypothetic echinocandin acylase, designated as ECEA. ECEA from the bacterium *Streptomyces* sp. SY1965 was previously isolated from sediment collected from the Mariana Trench and was identified as a producer of antifungal secondary metabolites [16]. SY1965 was then tested for its deacylation activity of echinocandins.

### 2.2. Deacylation Activity of the Hadal Bacterium Streptomyces sp. SY1965

**Figure 3:**
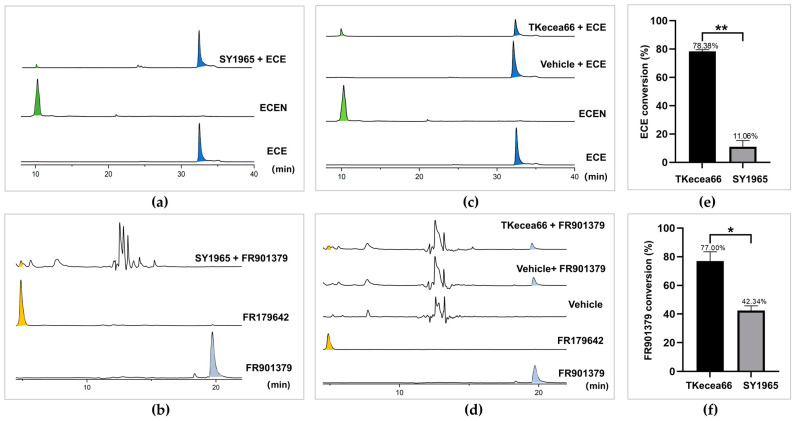
Whole-cell deacylation of ECE (blue shading) and FR901379 (pale blue shading) by the hadal bacterium *Streptomyces* sp. SY1965 and the recombinant echinocandin E acylase (ECEA) overproduction strain (TKecea66) to produce ECEN (green shading) and FR179642 (yellow shading), respectively. Bioconversion of ECE (**a**) and FR901379 (**b**) by SY1965; bioconversion of ECE (**c**) and FR901379 (**d**) by TKecea66; comparison of the conversion ratio of ECE in 12 h (**e**) and FR901379 in 3 h (**f**) by SY1965 and TKecea66, respectively. ECE: echinocandin E standard; ECEN: ECE nucleus standard; vehicle: *S. lividans* TK24 transferred with an empty pSET152 vector. Panels (**a**,**c**) show the EIC chromatograms from LC-TOFMS profiling by extracting the pseudo-molecular ions of ECE at *m*/*z* 734 and 756, and ECEN at *m*/*z* 996 and 1018, respectively. Panels (**b**,**d**) show the HPLC chromatograms at 210 nm. The * and ** in (**e**,**f**) denote statistical significances at *p* < 0.01 and *p* < 0.001, respectively.

Assuming that the gene encoding ECEA is expressed constitutively in *Streptomyces* sp. SY1965, it can be directly used for the whole-cell biotransformation of ECE to produce the ECEN. Since the hypothetic ECEA protein was prioritized using FR901379 acylase as the probe, it is also supposed to be able to catalyze the deacylation chemistry in FR901379, the parent compound of micafungin. To test this hypothesis, both ECE and FR901379 were used as substrates for deacylation biotransformation assays. After 12 h of incubation, HPLC and EIC (extracted ion chromatogram) peaks corresponding to the hydrolyzed product of the substrates were detected, albeit in limited titers. In contrast, the host strain transferred with an empty vector (vehicle) showed only background metabolites. (Figure 3a,b and Figure S1). The background metabolic profiles of the host strain are shown in Figure 3d (vehicle). LC-TOFMS provided proton and sodium adducts of the ECEN at *m*/*z* 734.3710 and 756.3532 (Figure S2), respectively, which confirmed the hydrolysis (deacylation) activity of strain SY1965. For the reaction mixtures using FR901379 as the substrate, the deacylated product FR179642 was observed at *m*/*z* 857.3525 [M + H-SO_3_]^+^ and 839.3430 [M + H-SO_3_-H_2_O]^+^ (Figure S2), again showing successful cleavage of the fatty acid chain of the substrate by SY1965. These observations suggest that the hypothetic ECEA in SY1965 is capable of hydrolyzing the fatty acid side chain from echinocandin’s natural products. The poor activity may stem from either intrinsic structure reasons or its low production level. In either case, microbial biotransformation efficiency can be improved via the heterologous overproduction of ECE acylase.

### 2.3. Engineering of Streptomyces Lividans TK24 for the Heterologous Production of Recombinant ECEA

To improve the efficiency of the deacylation reaction, we then considered constructing a recombinant strain in *Streptomyces lividans* TK24 to improve the production of the ECEA protein. The exconjugant TKecea66 carries the *ecea* overexpression cassette at the *attB* site of its chromosome (Figures S3–S6, Tables S3 and S4). It was subsequently used for ECEA production and whole-cell biotransformation of ECE and FR901379. The SDS-PAGE gel analysis of the Ni-NTA purified proteins revealed two bands at ~63 and ~19 kD (Figure S7). The two bands represent the characteristic α- and β-subunits derived from the autocatalyzed activation of a single precursor peptide of ECEA. This feature actually represents a characteristic hallmark of the Ntn (*N*-terminal nucleophile)-hydrolase superfamily of proteins, including the known ECBDs, FR901379 acylases, and penicillin/aculeacin A acylases. This result showed that the mature protein of ECEA was successfully produced in the heterologous host. The autoproteolytic cleavage of the nascent peptide supports the fact that ECEA is a member of the Ntn-hydrolase superfamily.

### 2.4. Deacylation of Echinocandin E using the Recombinant Strain TKecea66

The phenotype-verified (Figures S5 and S6) TKecea66 was then tested for its deacylation activity. The TKecea66 cultures (24 h) were used for the whole-cell biotransformation of ECE. After incubation for 12 h, the LC-TOFMS and HPLC profiling of the reaction mixtures showed significant consumption of the ECE substrate. Meanwhile, a much more polar product was observed at a significantly shorter retention time (reverse-phase chromatography), corresponding to the ECEN standard (Figure 3c and Figure S1). This is in accordance with the fact that the cleavage of the hydrophobic fatty acid moiety leads to a polar cyclic peptide nucleus (Figure 1). The high-resolution mass ions at *m*/*z* 734.3715 [M + H]^+^ and 756.3539 [M + Na]^+^, observed with LC-TOFMS, further confirmed the polar product as the deacylated ECE, namely the ECEN (Figure S2). While the host strain transferred with only empty vectors (the vehicle) showed no deacylation activity, a total of 78.38% of ECE substrate was converted into the ECEN in 12 h. In contrast, the native producer of ECEA (SY1965) showed only 11.06% conversion of the substrate, representing a 7.09-fold improvement in its relative activity (Figure 3e). These findings demonstrated that the recombinant strain and the ECEA protein it produces are capable of highly efficient ECE deacylation.

### 2.5. Deacylation of FR901379 using the Recombinant Strain TKecea66

As observed in the ECE biotransformation reactions, FR901379 was also successfully converted into the cyclic hexapeptide FR179642 (Figure 3d). The production of FR179642 was also confirmed via a high-resolution mass experiment by finding the pseudo molecular ions at *m*/*z* 839.3410 [M + H-SO_3_-H_2_O]^+^ and 857.3514 [M + H-SO_3_]^+^ (Figure S2). It took only 3 h to hydrolyze 77.00% of the substrate (0.17 mM), which was 1.82-fold more efficient than *Streptomyces* sp. SY1965 (Figure 3f). Apparently, FR901379 is a much better substrate than ECE. The difference between the two substrates may stem from two reasons. The EFI-EST tools prioritized enzymes similar to the probe FR901379 acylase, which favors FR901379 over ECE as their substrate. The other reason is very likely associated with the availability of the substrate in the reaction mixtures. Considering that ECE is a tetradeoxy analog of ECB, the modifications significantly increased the hydrophobicity of ECE. In the biotransformation assays, we could hardly increase the concentration of ECE above 0.1 mM. It is possible that the ECEA protein was not saturated by ECE in the reaction mixtures, leading to a catalytic rate lower than its maximum velocity. Therefore, the limited solubility of ECE could be the major reason for the relatively slower conversion activity.

### 2.6. Analysis of the Predicted Structure of ECEA

**Figure 4:**
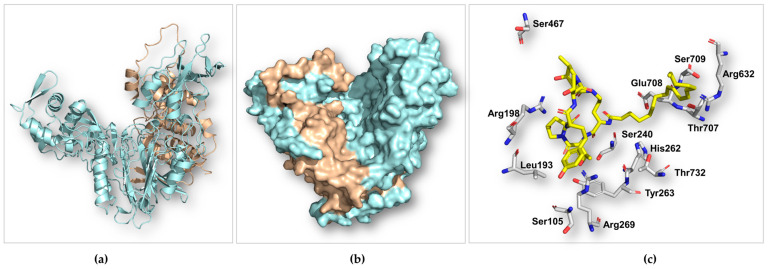
The predicted structure of ECEA generated using AlphaFold2. The ribbon (**a**) and surface (**b**) rendering of ECEA shows the distinct αββα fold (**a**) and the bowl-shaped catalytic pocket (**b**). The linker peptide (the 11 residues between E228 and S240) was manually removed in the surface representation of ECEA. The panel (**c**) displays the selected residues (gray) suggested to play a catalytical or significant binding role surrounding the substrate ECE (yellow). The shown structures are in the immature forms of ECEA built with a single molecule of the polypeptide. The α- (tint wheat) and β-subunit (pale cyan) are still connected by the linker peptide.

The multiple sequence alignment showed that ECEA is closely related to aculeacin A acylase, ECB acylases, and other representative members from the Ntn-hydrolase superfamily (Figure S8). The Ntn-hydrolase proteins adopt unique autocatalyzed chain cleavages to form a single-domain heterodimer. The structure of ECEA calculated using AlphaFold2 [17] is not in its mature form, which consists of an α- and a β-subunit (Figure 4a,b). Nonetheless, it still showed a high resemblance with Ntn-hydrolase proteins (Figure S9). ECEA demonstrated a distinctive architectural αββα fold (also known as an Ntn fold, Figure 4a). This highly conserved fold confers both stability and catalytic functionality to the proteins within the Ntn-hydrolase superfamily [18,19]. The Ser240 of ECEA is superimposed well with the *N*-terminal serine residue (Ser170) in the β-subunit of cephalosporin acylase (CPC, PDB entry: 1JVZ) (Figure S9). The Ser170 in CPC critically acts as both the proton donor and the nucleophile during self-cleavages and amide bond hydrolysis (Figure S9). Apparently, the autoactivation and catalytic mechanism employed by ECEA should be identical to other members of the Ntn-hydrolase superfamily. Specifically, two sequential autoproteolytic cleavages activate the nucleophilic residue (Ser240), and deacylation catalyzed by the serine residue proceeds via a covalent acyl-enzyme intermediate [20].

The immature ECEA protein is a single-chain molecule and retains the linker peptide (the 11 residues between Glu228 and Ser240) between the α- and β-subunits. The linker peptide sits in the catalytic pocket, blocking substrate binding. As a result, attempts to dock ECE, ECB, or glutaryl 7-aminocephalosporanic acid (GL-7ACA) to ECEA were not successful. Alternatively, we docked ECE to the mature form CPC to investigate the binding profiles of ECE with ECEA. As illustrated in Figure S10, ECE is situated at the bottom of the bowl-shaped binding pocket of CPC, with the hexapeptide predominantly binding to the α-subunit and the fatty acid chain binding to the β-subunit. The catalytically critical serine is positioned adjacent to the *N*-acyl amide bond (Figure 4c, Figures S9 and S10), suggesting the same catalytic mechanism adopted by both enzymes. The homotyrosine residue of ECE interacts with three aromatic residues via π-π stacking in CPC (Figure S10). In ECEA, it can be stabilized via π-π stacking from Tyr263, cation-π, and hydrophobic interactions from Arg269 to Leu193, respectively. Ser467 and Arg198 are also believed to be important in binding the cyclic peptide core of ECE (Figure 4c). In the CPC-ECE complex, the fatty acid side chain is stabilized by Phe529, Tyr598, and Arg632 (Figure S10). However, the corresponding aromatic residues are not available in ECEA, indicating a different binding mode of the acyl group in ECEA.

In summary, the predicted structure of ECEA reveals the characteristic Ntn fold, overall architecture, and catalytic mechanism shared by the Ntn-hydrolase superfamily. However, because of the limited sequence similarity within this superfamily, the exact binding interactions between ECE and ECEA remain to be fully elucidated. Future investigations employing site-directed mutagenesis and X-ray crystallography would be particularly significant in advancing our understanding of this enzyme.

## 3. Materials and Methods

### 3.1. Bioinformatic Analysis

The amino acid sequence of FR901379 acylase (Uniport: Q75RX8, GenBank: AB158476.1) was submitted as a seed sequence to the EFI-EST web tool (https://efi.igb.illinois.edu, accessed on 7 August 2023) to BLAST the UniProt database with a negative log of an e-value of 5. The retrieved proteins were exported as a FASTA file from the EFI-EST web tool. In-house homologous proteins to FR901379 acylase were obtained by using BLAST for the whole genome sequences of deep-sea derived microorganisms. They were manually combined into a single FASTA file, which was then uploaded to EFI-EST web tools to obtain the similarities between sequence pairs (alignment score > 10^−5^) and calculate the edge values for the initial sequence similarity networks (SSNs). The resulting SNN was refined with alignment length and alignment score thresholds of 740 and 260 (~60% identity), respectively. The final SNN was recreated and visualized using Cytoscape, showing sequence identities in the range of 54.61~100%. FR901379 acylase and the known ECB acylase (G9B7M9) were used as indicators of new echinocandin acylases. Proteins clustered with these functionally characterized acylases were inspected for their origins and function annotations. MEGA (version: 11.0.14) was employed for multiple sequence alignments of the hypothetic ECEAs, cephalosporin C acylase, FR901379 acylase, and ECBDs using the MUSCLE algorithm. The structure of ECEA was predicted using AlphaFold2 from AlphaFold Colab. The AutoDockTools (1.5.7) were used to prepare and run the molecular docking simulations using the genetic algorithm. Protein structures were visualized, analyzed, and rendered using PyMOL.

### 3.2. Strains and Reagents

The in-house hadal bacteria, including *Streptomyces* sp. SY1965, were isolated either from hadal sediments (MTD11000, November 2018) collected from the Mariana Trench (11°20′ N, 142°11.5′ E) at a depth of 11,000 m [16] or from the Kermadec Trench (30°47′ S, 177°11′ W) at a depth of 9779 m (November 2022). *Streptomyces* sp. SY1965 was maintained on MS agar medium (20 g/L soybean flour, 20 g/L mannitol, CaCO_3_ 3.0 g/L, and 20 g/L agar) prepared with artificial seawater (2.35% NaCl, 1.078% MgCl_2_, 0.147% CaCl_2_, 0.4% Na_2_SO_4_, 0.068% KCl, 0.0196% NaHCO_3_, 0.0026% borax, 0.00003% EDTA, and 0.003% Na_2_SiO_3_). *S. lividans* TK24 and its engineered mutants for ECEA production were maintained on MS agar medium. Liquid TSB (Tryptic Soy Broth) and SGGP [21] media were used for seed cultures and protein production cultures, respectively. *Aspergillus nidulans* LO8030-5.1 was constructed and cultured as described previously [10]. The other strains, including *E. coli* DH5α and the plasmids used for cloning and subcloning, are listed in Supplementary Table S2. They were maintained and used as described elsewhere [22]. ECE was prepared using an engineered strain of *A. nidulans* [10]. FR901379 and FR179642 were purchased from Aladdin (Shanghai, China). The primers used in this study (Table S3) were synthesized by Sunya (Hangzhou, China). The enzymes for cloning and gene editing were purchased from TAKARA and NEB, and we followed the protocols provided by the manufacturer. The DNA gel extraction and plasmid preparation kits were purchased from Sangon (Shanghai, China). The DNA sequencing was conducted by Sunya (Hangzhou, China). Regular chemicals and biochemical and media components were purchased from regular commercial sources, including Sinopharm (Beijing, China), TransGen Biotech (Beijing, China), Oxoid (Beijing, China), and Macklin (Shanghai, China).

### 3.3. HPLC and LC-MS Analyses

LC-MS was performed on an Agilent 1260 Infinity LC system coupled to a 6230 mass spectrometry machine consisting of a high-resolution TOF (time of flight) mass analyzer and an ESI (electrospray ionization) ion source. Analytic HPLC was performed on a SHIMAZU LC-20 machine equipped with a DAD detector. An Agilent PrepStar system was used for the semi-preparative HPLC analysis over a COSMOSIL C18-MS-II (5 μm, 10 × 250 mm). The enzymatic products were identified with the LC-TOFMS system in the positive mode. A YMC-ODS-A C18 column (250 × 4.6 mm, 5 µm) was used for all the HPLC and LC-MS analyses, with a constant column temperature of 30 °C. The following elution method was used for the HPLC and LC-MS analysis of ECE: mobile phase A: H_2_O containing 0.5% (*v*/*v*) TFA; mobile phase B: acetonitrile; elution program (B%): 0–10 min, 15–40%; 10–40 min, 40–100%; flow rate: 0.6 mL/min; injection volume: 20 μL. For the analytic HPLC analysis of FR901379, the following method was used (B%): 0–5 min, isocratic elution, 5%; 5–10 min, 5–55% B; 10–25 min, 55–100%; 25–33 min, 100%. The flow rate was 1.0 mL/min, and the injection volume was 20 μL. The reaction mixtures were quenched with an equal volume of methanol and centrifuged at 13,000× *g* for 15 min. The supernatants were then transferred to autosampler vials for LC-TOFMS or HPLC analysis without further purification. The semi-preparative purification of ECE was conducted via isocratic elution with 85% MeOH (3 mL/min) with UV detection at 222 nm. The ECEN was purified via isocratic elution with 16% ACN (3 mL/min) with UV detection at 210 nm.

### 3.4. Heterologous Expression of the ECE Acylase Gene in S. lividans TK24

The DNA fragment encoding ECEA was amplified from the genomic DNA of *Streptomyces* sp. SY1965 (Figure S3). A 6 × His tag was introduced to the C-terminal of ECEA by overhanging the primers to facilitate protein purification. The recombinant ECEA contained a native signal peptide directing its secretory and extracellular production. Overlap extension PCR was used to assemble the structure gene of *ecea* (including the signal peptide and the 6 × His tag) and the lambda T0 terminator (Tables S3 and S4). The resulting DNA fragment and the pSET152 vector were treated with the restriction enzymes AflII and EcoRI. We then ligated the gel-purified vector backbone and the insert with T4 DNA ligase. We made sure that the ECEA gene was controlled by the strong constitutive *kasO* promoter on the backbone (Figure S4). The ligation mixture was then transformed into chemically competent *E. coli* DH5α via heat shock. The transformants were allowed to grow on a medium containing 50 μg/mL of apramycin. The plasmid DNA was rescued from several colonies and verified by sequencing across the entire insert DNA. The correct recombinant plasmids were then transformed into *E. coli* ET12567 (PUZ8002). The intergeneric conjugal transfer [23] of the plasmid to *S. lividans* TK24 allowed the integration of the expression cassette to the *attB* site of the chromosome. The phenotype of the exconjugants was verified via PCR amplification of the expression cassette (Figures S5 and S6).

### 3.5. Protein Production and Purification

The ECEA production strain was cultured in 150 mL of SGGP medium for 72 h. The fermentation broth was then centrifuged at 4000× *g* 4 °C for 30 min. The supernatant was passed through a 0.45 μm NY filter (Shimadzu, Shanghai, China). The filtrate was loaded to a Ni-NTA (TransGen Biotech, Beijing, China) equipped with an ÄKTA start protein purifying system (GE, Boston, MA, USA). The elution buffer used for ECEA purification was 100 mM Tris-HCl (pH 8.0), containing 300 mM imidazole. The chromatographic peaks corresponding to ECEA were collected and concentrated using an ultrafilter tube (Sigma-Aldrich, Shanghai, China). The chromatographic fractions and crude protein samples (cell pellets) were analyzed using an SDS-PAGE gel and Coomassie staining (Figure S7).

### 3.6. Preparation of ECE and the ECEN

The new echinocandin congener ECE was produced by *A. nidulans* LO8030-5.1, which overexpresses an engineered echinocandin B biosynthetic gene cluster [10]. The mycelium from ten liters of fermentation broth was collected and exhaustively extracted using methanol with sonication at 25 °C. The resulting crude extract was subject to semi-preparative HPLC eluted isocratically with 85% MeOH over a COSMOSIL C18-MS-II column (5 μm, 10 × 250 mm). The purified ECE (60.2 mg) was used as a substrate of ECE and a standard for the HPLC and LC-MS analyses.

The ECEN was isolated from the biotransformation cultures of TKecea66 using ECE as the substrate. Briefly, the 2 L cultures of TKecea66 were cultured in SGGP medium for 24 h, and 50 mg of the ECEN was added for whole-cell deacylation. We continued the fermentation for another 24 h. Resin capture of the product was performed with 20 g of HP20 (Diaion^®^, Mitsubishi Chemical, Tokyo, Japan). After removing the impurities with 30% MeOH, the crude ECEN (120 mg) was eluted with 60% MeOH. The ECEN was then purified via semi-preparative HPLC using the COSMOSIL C18-MS-II column (5 μm, 10 × 250 mm) via isocratic elution with 16% acetonitrile containing 0.01% TFA.

### 3.7. Biotransformation Assay

The ECEA production strain was cultured for 24 h in SGGP medium at 28 °C with shaking at 200 rpm. For each 1 mL of culture, ECE or FR901379 was added to final concentrations of 0.1 and 0.17 mM, respectively. We continued to culture the ECE biotransformation mixtures for 12 h, and the FR901379 cultures were cultured for 3 h. The enzymatic reactions were quenched with 1 mL of methanol. The bioconversion ratios were calculated according to the HPLC peak areas of the substrates (3 replicates per experiment). GraphPad Prism 9 software was used for the statistical analysis. A paired Student’s *t* test was used for evaluating statistically significant differences between the conversion ratios regarding different strains using different substrates.

## 4. Conclusions

In the present paper, we described a novel echinocandin acylase, designated as ECEA, employing EFI-EST web tools. The gene encoding ECEA was identified from a Mariana Trench-derived bacterium, *Streptomyces* sp. SY1965. The subsequent heterologous expression of the *ecea* gene within *S. lividans* TK24 generated the mutant TKecea66, which exhibited a notably enhanced capacity for FR901379 and a novel echinocandin analog, ECE. Comparative analyses between TKecea66 and its parental strain SY1965 revealed an impressive 7.08- and 1.30-fold increase in the conversion ratios of ECE and FR901379, respectively. These findings underscore the potential of TKecea66 and ECEA in the development of novel echinocandin-based antifungal therapeutics. Our results also demonstrate the efficacy of the genomic enzymology approach in prioritizing new enzymatic activities. Furthermore, ECEA casts further light on the rich biodiversity within the hadal ecosystems for new function discovery.

## Data Availability

The original data presented in the study are included in the article/Supplementary Material; further inquiries can be directed to the corresponding author.

## Supplementary Materials

The following supporting information can be downloaded at: https://www.mdpi.com/article/10.3390/md22050212/s1, Table S1: Marine derived proteins clustered with the probe FR9013179 acylase in SNN generated by EFI-EST; Table S2: Strains and plasmids used in this study; Table S3: Primers used in this study; Table S4: DNA fragments for assembling the *ecea* expressing cassette; Table S5: Amino acid sequence of ECEA including the signal peptide; Figure S1: HPLC profiles showing the deacylation activity of ECE and FR901379 by the hadal bacterium *Streptomyces* sp. SY1965 (a, c) and the recombinant echinocandin E acylase (ECEA) overproducing strain (TKecea66)(b, d); Figure S2: High-resolution mass spectra showing the production of deacylated products of ECEN and FR179642 from substrates ECE and FR901379, respectively; Figure S3: Construction of the *ecea* gene overexpression plasmid for intergeneric conjugation. The DNA fragment (2415 bp) amplified from the genomic DNA of *Streptomyces* sp. SY1965 (a); PCR verification of recombinant plasmid pSET-*kasO*p * ECEA (b). M: DNA Marker; left gel: lanes 1-9 are *E. coli* DH5α transformants; Lane 9 represents the correct construct; Figure S4: Plasmid map of pSET-*kasOp** ECEA. The *ecea* gene expression cassette consists of the *KasO*p* (with RBS) promoter, structure gene of *ecea*, 6×His tag and the Lambda T0 terminator; Figure S5: Schematic diagram showing the integration site of the *ecea* gene and PCR verification of the expression cassette on the chromosome of the recombinant strain, *Streptomyces lividans* TKecea66; Figure S6: PCR verification of the genotype of *S. lividans* TKecea66. Agarose (1%)/TAE gel electrophoresis showing the upstream (a) and downstream (b) junctions flanking the *ecea* gene. M: DNA Marker; lines 1-8: exconjugants; line 9: *S. lividans* TK24; line 10: exconjugant with empty plasmid; Figure S7: SDS-PAGE analysis of the ECEA produced by the recombinant strain *Streptomyces lividans* TKecea66. M: Marker; Lane 1: supernatant of *S. lividans* TK24; lane 2: supernatant of TKecea66; lane 3: *S. lividans* TK24 transferred with an empty pSET152 vector; lane 4: fractions eluted with buffer containing 20 mM imidazole; Lane 5: fractions eluted with buffer containing 50 mM imidazole; line 6: fractions eluted with buffer containing 100 mM imidazole; line 7: fractions eluted with buffer containing 300 mM imidazole; line 8: fractions eluted with buffer containing 500 mM imidazole; Figure S8: Multiple sequence alignment of ECEA, hypothetic ECE acylases (blue), known proteins (red) and a cephalosporin C acyltransferase (CAD, GenBank accession number: AF251710.1, green). Key catalytic sites marked with red stars and active pocket residues marked with triangles. echinocandin B acylase gene (GenBank accession numbers: BD226911, AB158476.1, HM851181, D10610); Figure S9: Comparison of the structures of ECEA (green) and CPC (orange, PDB code: 1JVZ), RMSD= 2.469. ECEA was generated by Alphafold2; Figure S10: The structure (a) of ECE and CPC protein docking that α-subunit colored by light blue and β-subunit colored by light pink, and shown the key residues (cyan) surrounding substrate of ECE (yellow) within 6 Å (b).

## References

[1] Denning D.W. (2003). Echinocandin antifungal drugs. Lancet.

[2] Perlin D.S. (2015). Mechanisms of echinocandin antifungal drug resistance. Ann. N. Y. Acad. Sci..

[3] Antinori S., Milazzo L., Sollima S., Galli M., Corbellino M. (2016). Candidemia and invasive candidiasis in adults: A narrative review. Eur. J. Intern. Med..

[4] Szymański M., Chmielewska S., Czyżewska U., Malinowska M., Tylicki A. (2022). Echinocandins—Structure, mechanism of action and use in antifungal therapy. J. Enzyme Inhib. Med. Chem..

[5] Syed Y.Y. (2023). Rezafungin: First Approval. Drugs.

[6] Shao L., Li J., Liu A., Chang Q., Lin H., Chen D. (2013). Efficient Bioconversion of Echinocandin B to Its Nucleus by Overexpression of Deacylase Genes in Different Host Strains. Appl. Environ. Microbiol..

[7] Zou S.-P., Han X., Zhu H.-Y., Sheng Q., Tang H., Liu Z.-Q., Zheng Y.-G. (2021). Functional expression of an echinocandin B deacylase from *Actinoplanes utahensis* in *Escherichia coli*. Int. J. Biol. Macromol..

[8] Kreuzman A.J., Hodges R.L., Swartling J.R., Pohl T.E., Ghag S.K., Baker P.J., McGilvray D., Yeh W.K. (2000). Membrane-associated echinocandin B deacylase of *Actinoplanes utahensis*: Purification, characterization, heterologous cloning and enzymatic deacylation reaction. J. Ind. Microbiol. Biotechnol..

[9] Ueda S., Shibata T., Ito K., Oohata N., Yamashita M., Hino M., Yamada M., Isogai Y., Hashimoto S. (2011). Cloning and expression of the FR901379 acylase gene from *Streptomyces* sp. no. 6907. J. Antibiot..

[10] Yu X., Jiang Q., Chen X., Shu H., Xu Y., Sheng H., Yu Y., Wang W., Keller N.P., Xu J. (2023). Unnatural tetradeoxy echinocandins produced by gene cluster design and heterologous expression. Org. Biomol. Chem..

[11] Polderman-Tijmes J.J., Jekel P.A., de Vries E.J., van Merode A.E., Floris R., van der Laan J.M., Sonke T., Janssen D.B. (2002). Cloning, sequence analysis, and expression in *Escherichia coli* of the gene encoding an alpha-amino acid ester hydrolase from *Acetobacter turbidans*. Appl. Environ. Microbiol..

[12] Zallot R., Oberg N., Gerlt J.A. (2019). The EFI Web Resource for Genomic Enzymology Tools: Leveraging Protein, Genome, and Metagenome Databases to Discover Novel Enzymes and Metabolic Pathways. Biochemistry.

[13] Oberg N., Zallot R., Gerlt J.A. (2023). EFI-EST, EFI-GNT, and EFI-CGFP: Enzyme Function Initiative (EFI) Web Resource for Genomic Enzymology Tools. J. Mol. Biol..

[14] Gerlt J.A., Bouvier J.T., Davidson D.B., Imker H.J., Sadkhin B., Slater D.R., Whalen K.L. (2015). Enzyme Function Initiative-Enzyme Similarity Tool (EFI-EST): A web tool for generating protein sequence similarity networks. Biochim. Biophys. Acta.

[15] Saito R., Smoot M.E., Ono K., Ruscheinski J., Wang P.-L., Lotia S., Pico A.R., Bader G.D., Ideker T. (2012). A travel guide to Cytoscape plugins. Nat. Methods.

[16] Yi W., Qin L., Lian X.-Y., Zhang Z. (2020). New Antifungal Metabolites from the Mariana Trench Sediment-Associated Actinomycete *Streptomyces* sp. SY1965. Mar. Drugs.

[17] Bryant P., Pozzati G., Elofsson A. (2022). Improved prediction of protein-protein interactions using AlphaFold2. Nat. Commun..

[18] Oinonen C., Rouvinen J. (2001). Structural comparison of Ntn-hydrolases. Protein Sci..

[19] Linhorst A., Lübke T. (2022). The Human Ntn-Hydrolase Superfamily: Structure, Functions and Perspectives. Cells.

[20] Kim Y., Yoon K.-H., Khang Y., Turley S., Hol W.G.J. (2000). The 2.0 Å Crystal Structure of Cephalosporin Acylase. Structure.

[21] Yamamoto H., Maurer K.H., Hutchinson C.R. (1986). Transformation of *Streptomyces erythraeus*. J. Antibiot..

[22] Elbing K.L., Brent R. (2019). Recipes and Tools for Culture of *Escherichia coli*. Curr. Protoc. Mol. Biol..

[23] Mazodier P., Petter R., Thompson C. (1989). Intergeneric conjugation between *Escherichia coli* and *Streptomyces* species. J. Bacteriol..

