# Supplementary material for: A Hadal Streptomyces-Derived Echinocandin Acylase Discovered through the Prioritization of Protein Families
Source: Mar Drugs. 2024 May 7;22(5):212. doi: 10.3390/md22050212 (PMC11122479; doi:10.3390/md22050212)
Supplement: Supplementary file 1 [file marinedrugs-22-00212-s001.zip › marinedrugs-2973390-supplementary.pdf]

Supplementary Materials

A hadal *Streptomyces*-derived echinocandin acylase discovered through prioritization of protein families

Xuejian Jiang [1], Hongjun Shu [1], Shuting Feng [1], Pinmei Wang [1, 2], Zhizhen Zhang [1] and Nan Wang [1, 2,*]

Table of Contents

Table S1. Marine derived proteins clustered with the probe FR9013179 acylase in SNN generated by EFI-EST 3

Table S2. Strains and plasmids used in this study........ 3

Table S3. Primers used in this study. 4

Table S4. DNA fragments for assembling the *ecea* expressing cassette 4

Table S5. Amino acid sequence of ECEA including the signal peptide 5

Figure S1. HPLC profiles showing the deacylation activity of ECE and FR901379 by the hadal bacterium *Streptomyces* sp. SY1965 (a, c) and the recombinant echinocandin E acylase (ECEA) overproducing strain (TKecea66)(b, d). 7

Figure S2. High-resolution mass spectra showing the production of deacylated products of ECEN and FR179642 from substrates ECE and FR901379, respectively. 8

Figure S3. Construction of the *ecea* gene overexpression plasmid for intergeneric conjugation. The DNA fragment (2415 bp) amplified from the genomic DNA of *Streptomyces* sp. SY1965 (a); PCR verification of recombinant plasmid pSET-*kasO*p* ECEA (b). M: DNA Marker; left gel: lanes 1-9 are *E. coli* DH5α transformants; Lane 9 represents the correct construct. 9

Figure S4. Plasmid map of pSET-*kasOp** ECEA. The *ecea* gene expression cassette consists of the *KasO*p* (with RBS) promoter, structure gene of *ecea*, 6×His tag and the Lambda T0 terminator. 10

Figure S5. Schematic diagram showing the integration site of the *ecea* gene and

PCR verification of the expression cassette on the chromosome of the recombinant strain, *Streptomyces lividans* TKecea66.......10

Figure S6. PCR verification of the genotype of *S. lividans* TKecea66. Agarose (1%)/TAE gel electrophoresis showing the upstream (**a**) and downstream (**b**) junctions flanking the *ecea* gene. M: DNA Marker; lines 1-8: exconjugants; line 9: *S. lividans* TK24; line 10: exconjugant with empty plasmid.......11

Figure S7. SDS-PAGE analysis of the ECEA produced by the recombinant strain *Streptomyces lividans* TKecea66. M: Marker; Lane 1: supernatant of *S. lividans* TK24; lane 2: supernatant of TKecea66; lane 3: *S. lividans* TK24 transferred with an empty pSET152 vector; lane 4: fractions eluted with buffer containing 20 mM imidazole; Lane 5: fractions eluted with buffer containing 50 mM imidazole; line 6: fractions eluted with buffer containing 100 mM imidazole; line 7: fractions eluted with buffer containing 300 mM imidazole; line 8: fractions eluted with buffer containing 500 mM imidazole.......11

Figure S8. Multiple sequence alignment of ECEA, hypothetic ECE acylases (blue), known proteins (red) and a cephalosporin C acyltransferase (CAD, GenBank accession number: AF251710.1, green). Key catalytic sites marked with red stars and active pocket residues marked with triangles. echinocandin B acylase gene (GenBank accession numbers: BD226911, AB158476.1, HM851181, D10610). ...12

Figure S9. Comparison of the structures of ECEA (green) and CPC (orange, PDB code: 1JVZ), RMSD= 2.469. ECEA was generated by Alphafold2.......13

Figure S10. The structure (a) of ECE and CPC protein docking that α-subunit colored by light blue and β-subunit colored by light pink, and shown the key residues (cyan) surrounding substrate of ECE (yellow) within 6 Å (b).......13

References.......14

Table S1. Marine derived proteins clustered with the probe FR9013179 acylase in SNN generated by EFI-EST

Origin	UniProt ID
Unknown proteins of intertidal origins	A0A2S1I7W9<br>A0A1S2PKA0<br>A0A2M9LMP4<br>A0A1Q5BWW0<br>A0A1Q5GMV2
Unknown sponge co-epiphytic microbial proteins	A0A3N6H9K9<br>A0A3N6DWY1<br>A0A3G4VTM0<br>A0A101URM3
Unknown microbial proteins derived from seafloor sediments	A0A4R1DSC3<br>H0BNF0<br>A0A3S9PDQ3<br>A0A1V0UK48<br>A0A6I6WZB9<br>A0A941FGI5<br>A0A1E7LNN9<br>F3NT93
Hadal origin protein studied	ECEA*

*not a UniProt accession ID

Table S2. Strains and plasmids used in this study.

Strain / Plasmid	Function	Source
E. coli DH5α	Subcloning	In-house collection
E. coli ET12567(PUZ8002)	Intergeneric Conjugation	In-house collection
Streptomyces lividans TK24	Host for ECEA production	In-house collection
Streptomyces sp. SY1965	Cloning of the *ecea* gene and biotransformation of ECE/FR9013179	[15]
pSET-*kasOp***ecea*	Conjugal transformation plasmid	This study
Aspergillus nidulans LO8030-5.1	ECE production	[9]
Streptomyces lividans TKecea66	Biotransformation of ECE/FR9013179	This study

Table S3. Primers used in this study.

Primer	Nucleotide Sequence (5'-3')	Function
ECEA_F1	gccttaagatgacgttacgcaaccgtctgagactgc	*ecea* gene amplification
ECEA_R1	ctagtggtggtggtggtggtgccgccgctcgtgcac	*ecea* gene amplification
ECEA_F2	caccaccaccaccaccactagactccatctggatttgttc	Overlap integration
ECEA_R2	gctgaacggggggttcgtgcacaca	Overlap integration
ECEAyz_F	ttgtaaaacgacggccagtgccaagct	Genotype PCR verification
ECEAyz_R	atgcttccggctcgtatgttgtgtgg	Genotype PCR verification
pSL_F5	cacgttttcccaggtcagaagc	Genotype PCR verification
pSL_R5	atgctcaactaaagtggggcg	Genotype PCR verification
pSL_F7	gaccatcgtgatcggcgtgt	Genotype PCR verification
pSL_R7	gcgtcgagccgcataatcagg	Genotype PCR verification

Table S4. DNA fragments for assembling the *ecea* expressing cassette

Fragment	DNA sequence (5′-3′)
The coding sequence of *ecea*	ttgacgttacgcaaccgtctgagactgctcggggtcgccggtctcgccctgttcaccgtgtcggcctcgctgccgcc<br>ggccgccgcgtccggggccgggccacagcggcacccgtccggcggcgggctctccgccgtcatccggtacacg<br>gagtacggcattccgcacatcgtggcgaaggactacgcgcagctcggcttcggcaccggctgggcgcaggcgg<br>ccgaccaggtgtgcacgctggcggacggtttcctcacggtgcgcggggaacggtcgaggttcttcggcccggac<br>gccgccccggacttctcgctctcctcggcggcgacgaacctctccagcgatctgtacttccggggtgtccgggaca<br>gcggcacggtggagaagctgctgaaggtgcccgcgcccgccggtccgagccgggacgccaaggagacgatg<br>cgcgggttcgccgccgggtacaacgcgtggatccagcagaaccggatcaccgatccggcctgccggggcgcgt<br>cctgggtccgtccggtgacggcgctggacgtggcggcgcgcggctacgcgctggccgtgctcggcggtcaggg<br>gcgcggcatcgacggcatcacggcggcgcggccgccgaccgccgctcccccggcggcgggggtcacgcccga<br>ggaggcggcgacggcggccgagcggctgctgtcggcgcagaacgcggacatgggctccaacgcggtggcctt<br>cgacggctccaccacggtgaacgggcgcgggctgctgctcggcaacccgcactacccgtggcagggcggacgc<br>cgcttctggcagtcgcagcagaccattcccggtgagctgaacgtgtcgggtgcgtccctgctgggcgccacgacg<br>atctcgatcgggcacaacgcggatgtggcgtggagccatacggtcgccacgggcgtcacgctgaatctgcatca<br>gctgacgctggatccggccgacccgaccacgtatctggtggacgggaagcgggagcggatgacgaagcggac<br>ggtgagcgtcccggtgaagggcgcggctcccgtgacccgcacccagtggtggacgcgctacggcccggtcgtc<br>gactccatgggctcggcgctgccgttgccgtggacggcgagcacggcgtacgccctgaacgacccgaacgcga<br>cgaatctgcggatggcggacaccggtctgggcttcagcagggcccgctccacggcggacgtggaacgctcact<br>gcaccggaaccagggcatgccgtgggtgaacacgatcgcggcggaccgggcggggcactcgttcttcgcccag<br>tcgcaggtcctgccgaggatcacggacgagctggcggagcgctgctcgaccgcgctgggccgggccacgtatc<br>cggcgtccgggctggcggtgctggacggctcgcgcgaggactgcgcgctgggcagcgacccggacgcggtgc<br>agtcgggcatcttcggcccgagccggatgcccacgctcaagaaccggccgtacgtggagaactccaacgacagc<br>gcctggctgaccaacgcggagcagccgctgaccgggtacgagcgggtcttcggcacggtcgcgacgccccggt<br>cgatgcggacgcgtggcgcgatcgaggatgtcgcggagatggcggacaagggccgcctccgggtcgcggatc<br>tccagcggcagcagttcgccaaccgtgcgccggccggcgacctggccgcgtcggagacggcggggtggtgcg<br>cggcgctgccgggcggcacggccgtgggcacgggcggtacgccggtcgatgtgtcggaggcctgccgggtgc<br>tgcggcgctgggaccggagcgtggacagcgacagccggggcgcgctgctcttcgaccggttctggcggaggg<br>cctcggcggtgcccgccgccgagctgtggcggaccccgttcgacccggccgacccggtgcgcaccccgcgcggt<br>ctgaacaccgccgcgccgggtgtcggcacggcgctggcggacgcggtgacggagctgcgggcggcgggcat<br>cacgctcgacgcaccgctgggcaagcaccagttcgtggtccggaacggcaagcggctcccgatcggcggcggg<br>acggagtcgctgggcatctggaacaagaccgagcccgtgtggaacgcggcggcgggcggctatacggaggtg<br>tcgacgggctccagctacatccaggcggtcggctgggacgacagccgctgcccggtggcccggacgctgctga<br>cgtactcccagtcggagaacccgaagtcgccgcacttcagcgaccagacccggctgtacgcgggcgagcgctg

	ggtgacgtcccggttctgcgagaaggacatcgcccgctcgccggccctgcgggtggtgcgggtgcacgagcgg<br>cggtag
*Kas*Op*	tgttcacattcgaacggtctctgctttgacaacatgctgtgcggtgttgtaaagtcgtggccaggagaatacgacag<br>cgtgcaggactgggggagtt
6×His	caccaccaccaccaccac
lambda t0 terminator	actccatctggatttgttcagaacgctcggttgccgccgggcgttttttat

Table S5. Amino acid sequence of ECEA including the signal peptide

Amino acid sequence
LTLRNRLRLLGVAGLALFTVSASLPPAAASGAGPQRHPSGGGLSAVIRYTEYGIPHIVAKDY<br>AQLGFGTGWAQAADQVCTLADGFLTVRGERSRFFGPDAAPDFSLSSAATNLSSDLYFRGV<br>RDSGTVEKLLKVPAPAGPSRDAKETMRGFAAGYNAWIQQNRITDPACRGASWVRPVTAL<br>DVAARGYALAVLGGQGRGIDGITAARPPTAAPPAAGVTPEEAATAAERLLSAQNADMGS<br>NAVAFDGSTTVNGRGLLLGNPHYPWQGGRRFWQSQQTIPGELNVSGASLLGATTISIGHN<br>ADVAWSHTVATGVTLNLHQLTLDPADPTTYLVDGKRERMTKRTVSVPVKGAAPVTRTQ<br>WWTRYGPVVDSMGSALPLPWTASTAYALNDPNATNLRMADTGLGFSRARSTADVERSLH<br>RNQGMPWVNTIAADRAGHSFFAQSQVLPRITDELAERCSTALGRATYPASGLAVLDGSRE<br>DCALGSDPDAVQSGIFGPSRMPTLKNRPYVENSNDSAWLTNAEQPLTGYERVFGTVATPR<br>SMRTRGAIEDVAEMADKGRLRVADLQRQQFANRAPAGDLAASETAGWCAALPGGTAVG<br>TGGTPVDVSEACRVLRRWDRSVDSDSRGALLFDRFWRRASAVPAAELWRTPFDPADPVRT<br>PRGLNTAAPGVGTALADAVTELRAAGITLDAPLGKHQFVVRNGKRLPIGGGTESLGIWNK<br>TEPVWNAAAGGYTEVSTGSSYIQAVGWDDSRCPVARTLLTYSQSENPKSPHFSDQTRLYAG<br>ERWVTSRFCEKDIARSPALRVVRVHERR*

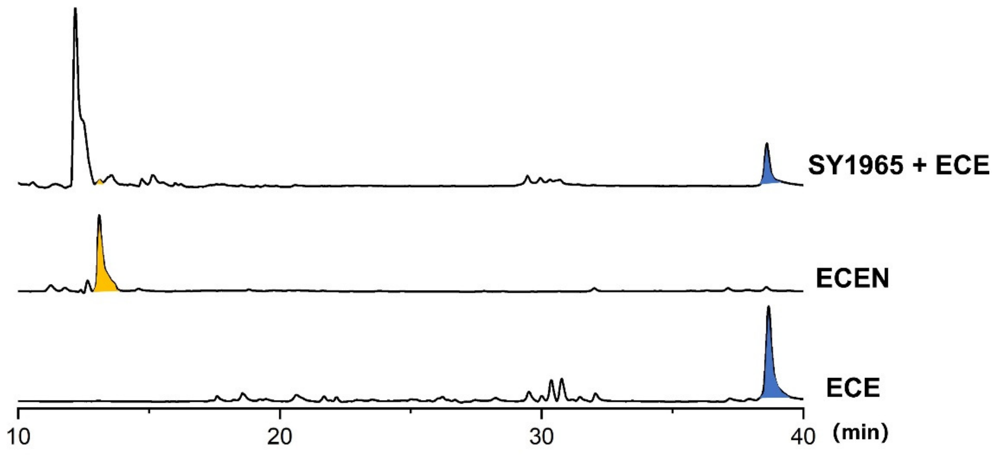
SY1965 + ECE
ECEN
ECE
10
20
30
40
(min)

(a)

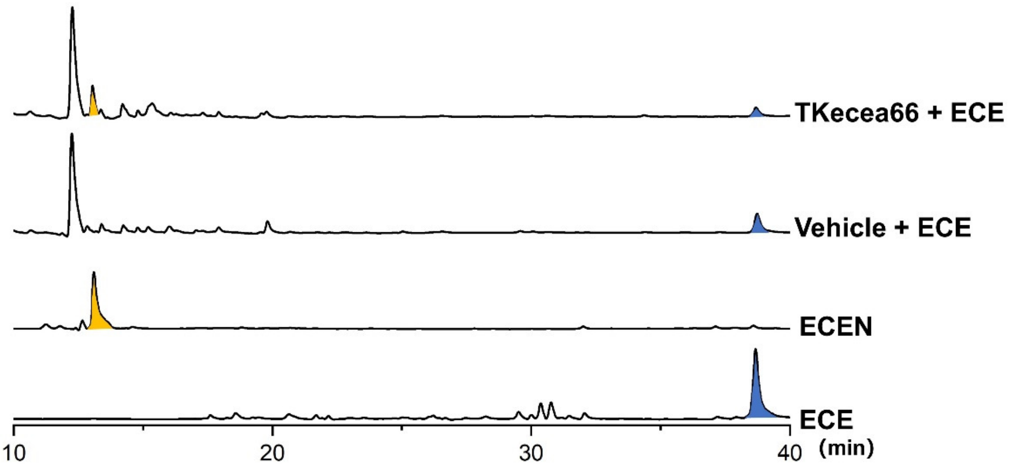
TKecea66 + ECE
Vehicle + ECE
ECEN
ECE
10
20
30
40
(min)

(b)

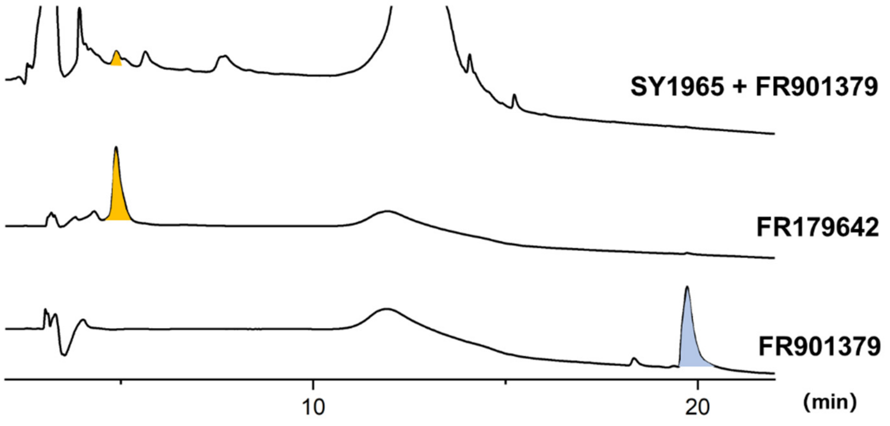
SY1965 + FR901379
FR179642
FR901379
10
20
(min)

(c)

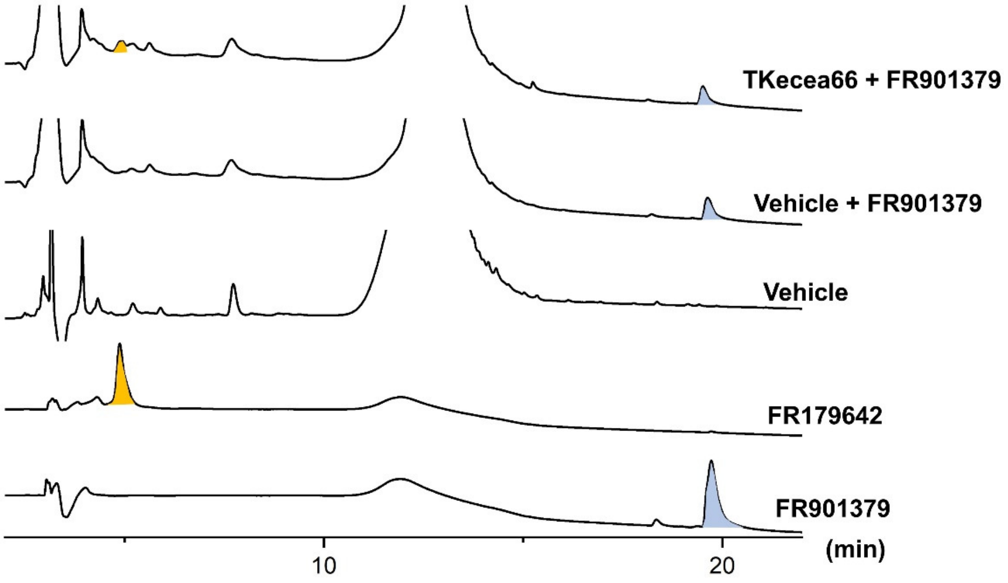

(d)

Figure S1. HPLC profiles showing the deacylation activity of ECE and FR901379 by the hadal bacterium *Streptomyces* sp. SY1965 (a, c) and the recombinant echinocandin E acylase (ECEA) overproducing strain (TKecea66)(b, d).

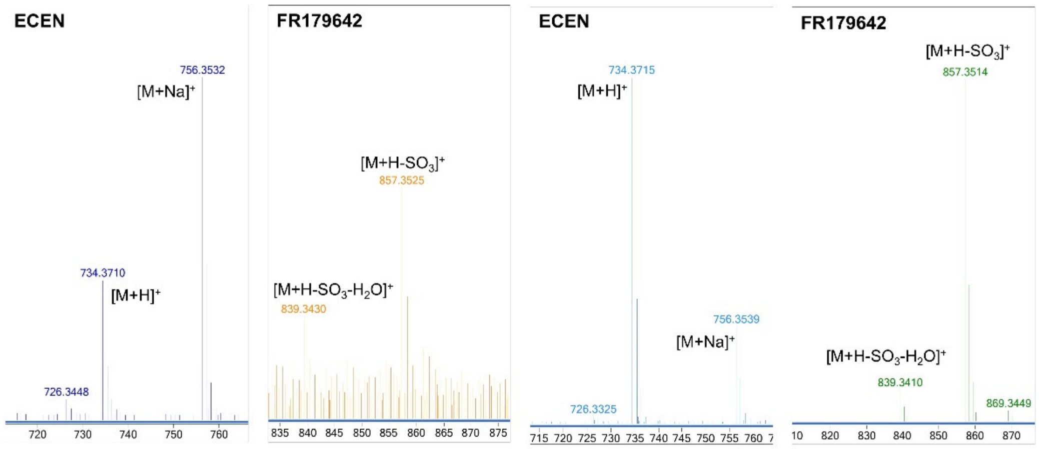

a b c d

Figure S2. High-resolution mass spectra identification the deacylated products of ECEN and FR179642 from ECE and FR901379 by *Streptomyces* SY1965 (**a**, **b**) and recombinant strain *S. lividans* TKecea66 (**c**, **d**).

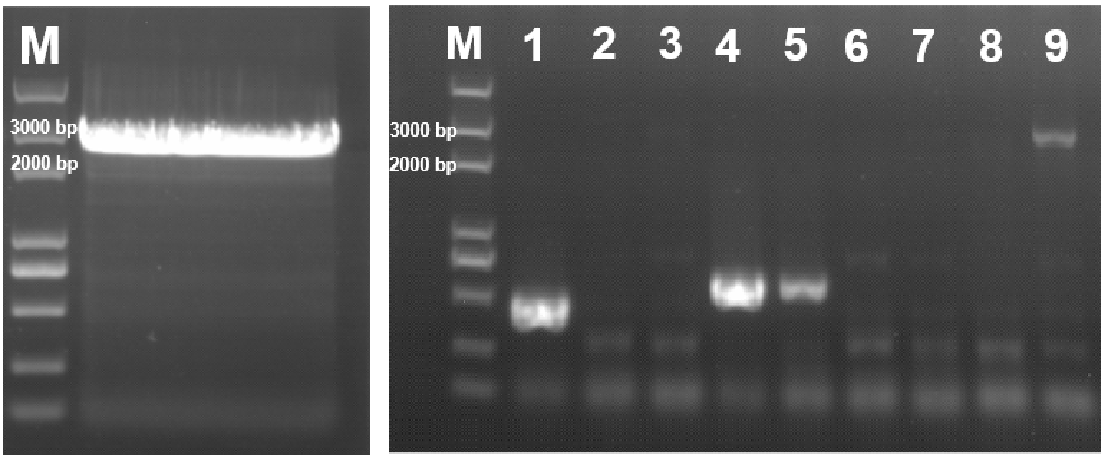

(a) (b)

Figure S3. Construction of the *ecea* gene overexpression plasmid for intergeneric conjugation. The DNA fragment (2415 bp) amplified from the genomic DNA of *Streptomyces* sp. SY1965 (a); PCR verification of recombinant plasmid pSET-*kasO*p**ecea* (b). M: DNA Marker; left gel: lanes 1-9 are *E. coli* DH5α transformants; Lane 9 represents the correct construct.

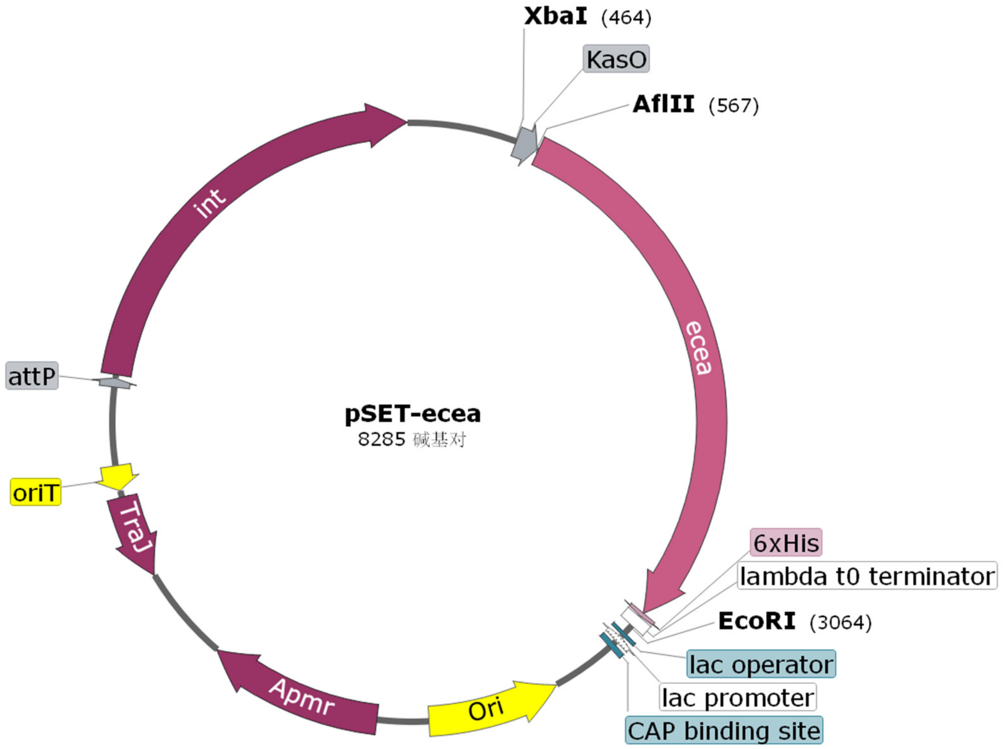

Figure S4. Plasmid map of pSET-*kasOp**ecea*. The *ecea* gene expression cassette consists of the *KasO*p* (with RBS) promoter, structure gene of *ecea*, 6×His tag and the Lambda T0 terminator.

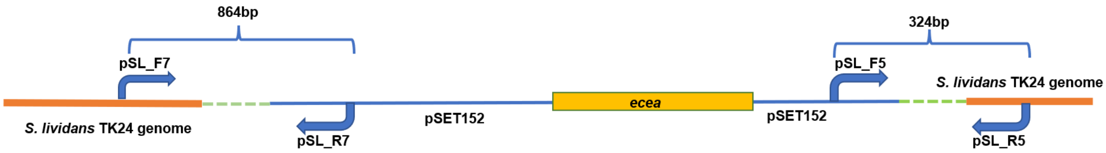

Figure S5. Schematic diagram showing the integration site of the *ecea* gene and PCR verification of the expression cassette on the chromosome of the recombinant strain, *Streptomyces lividans* TKecea66.

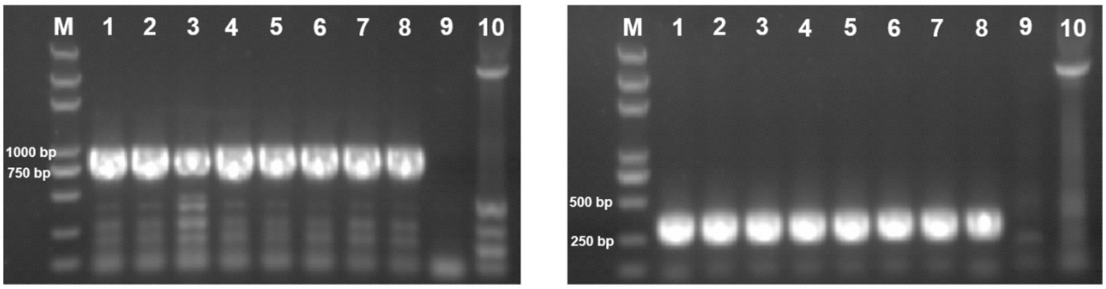

(a) **(b)**

Figure S6. PCR verification of the genotype of *S. lividans* TKecea66. Agarose (1%)/TAE gel electrophoresis showing the upstream (**a**) and downstream (**b**) junctions flanking the *ecea* gene. M: DNA Marker; lines 1-8: exconjugants; line 9: *S. lividans* TK24; line 10: exconjugant with empty plasmid.

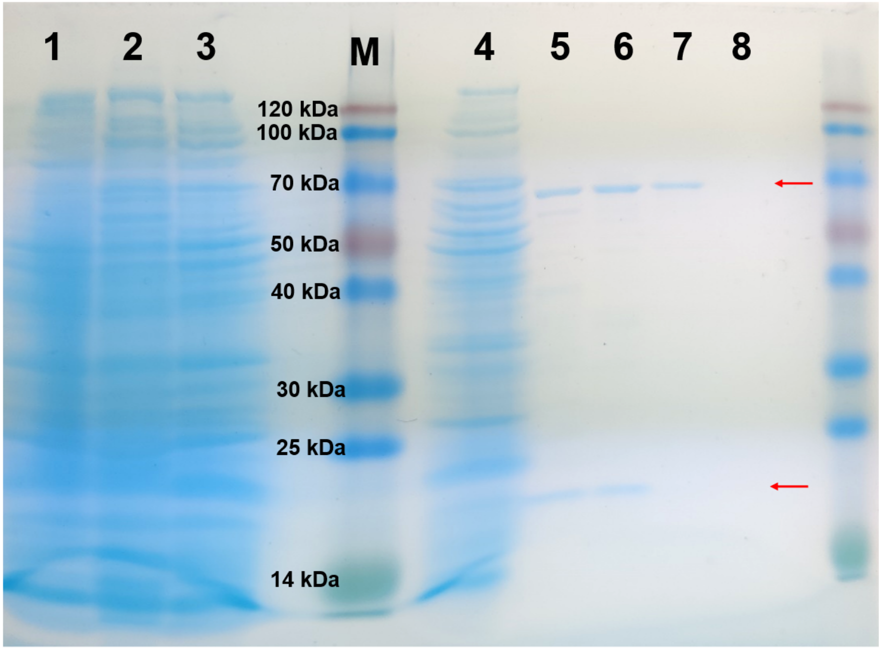

Figure S7. SDS-PAGE analysis of the ECEA produced by the recombinant strain *Streptomyces lividans* TKecea66. M: Marker; Lane 1: supernatant of *S. lividans* TK24; lane 2: supernatant of TKecea66; lane 3: *S. lividans* TK24 transferred with an empty pSET152 vector; lane 4: fractions eluted with buffer containing 20 mM imidazole; Lane 5: fractions eluted with buffer containing 50 mM imidazole; line 6: fractions eluted with buffer containing 100 mM imidazole; line 7: fractions eluted with buffer containing 300 mM imidazole; line 8: fractions eluted with buffer containing 500 mM imidazole.

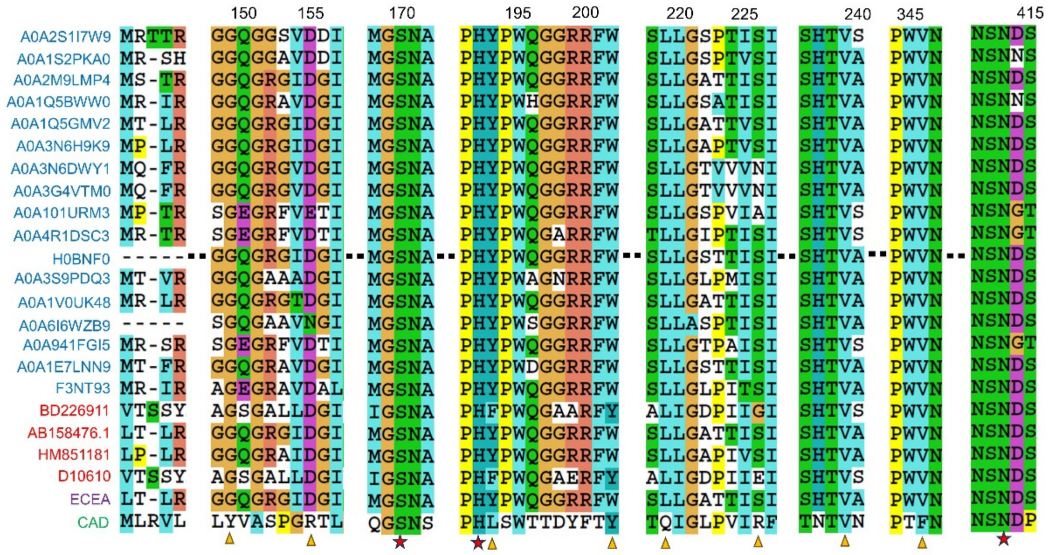

Figure S8. Multiple sequence alignment of ECEA, hypothetic ECE acylases (blue), known proteins (red) and a cephalosporin C acyltransferase (CAD, GenBank accession number: AF251710.1, green). Key catalytic sites marked with red stars and active pocket residues marked with triangles. echinocandin B acylase gene (GenBank accession numbers: BD226911, AB158476.1, HM851181, D10610).

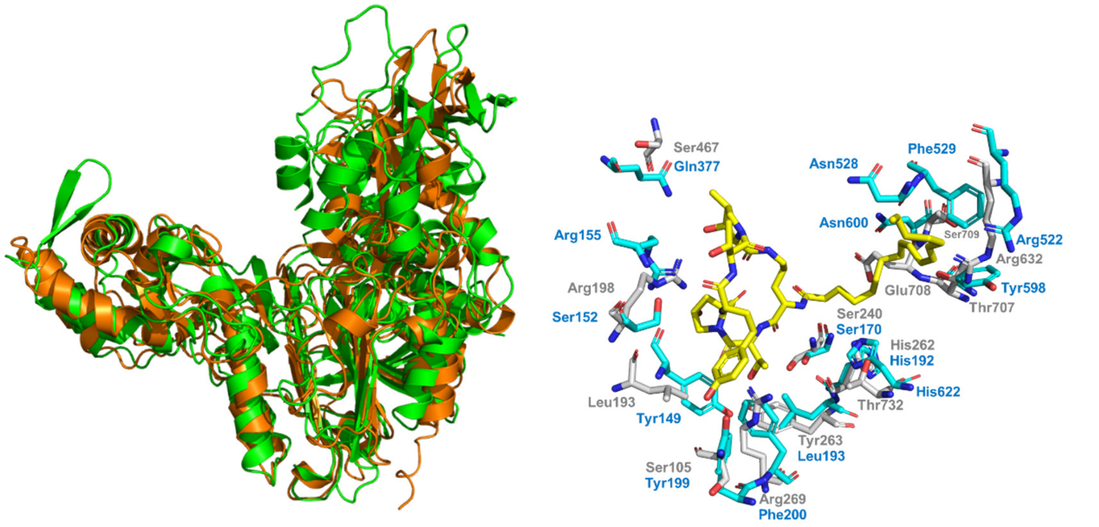

(a) **(b)**

Figure S9. Comparison of the structures of ECEA (green) and CPC (orange, PDB code: 1JVZ) (**a**) RMSD= 2.469. The catalytically critical Ser170 in CPC (cyan) superimposed with the Ser240 of ECEA (gray) (**b**). The structure of ECEA was generated by Alphafold2.

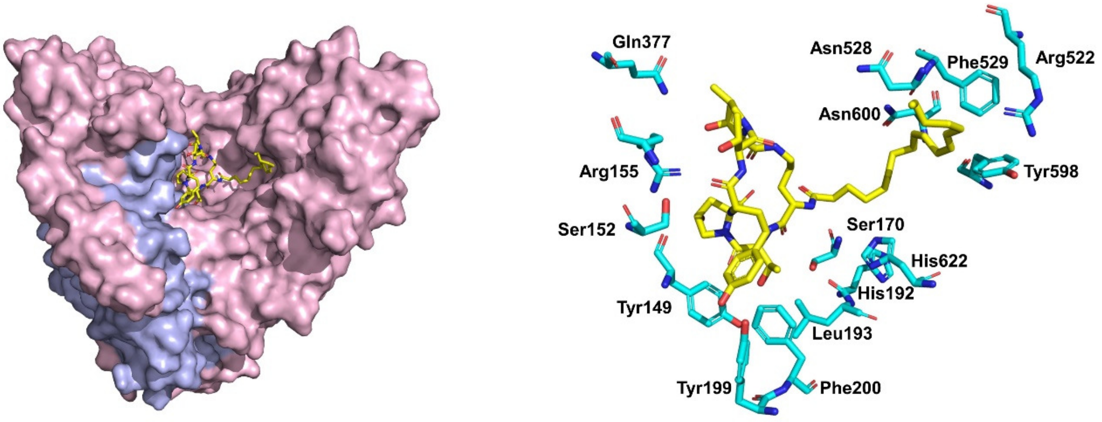

(a) **(b)**

Figure S10. The structure (a) of ECE and CPC protein docking that α-subunit colored by light blue and β-subunit colored by light pink, and shown the key residues (cyan) surrounding substrate of ECE (yellow) within 6 Å (b).

References

10. Yu, X.; Jiang, Q.; Chen, X.; Shu, H.; Xu, Y.; Sheng, H.; Yu, Y.; Wang, W.; Keller, N.P.; Xu, J. and Wang, P. Unnatural tetradeoxy echinocandins produced by gene cluster design and heterologous expression. *Org. Biomol. Chem.* **2023**, *21*, 3552-3556.
16. Yi, W.; Qin, L.; Lian, X.-Y. and Zhang, Z. New Antifungal Metabolites from the Mariana Trench Sediment-Associated Actinomycete *Streptomyces* sp. SY1965. *Mar. Drugs* **2020**, *18*, 385.
